# Report of Cases Treated at the Aberdeen New Dispensary

**Published:** 1827-12

**Authors:** Alexander Rainy

**Affiliations:** senior Pupil.


### THE LONDON
### Medical and Physical Journal.

No. 346, VOL. LVIII.] DECEMBER, 1827. [No. 18, *New Series*.

For many fortunate discoveries in medicine, and for the detection of numerous errors, the world is indebted to the rapid circulation of Monthly Journals; and there never existed any work, to which the Faculty, in Europe and America, were under deeper obligations, than to the Medical and Physical Journal of London, now forming a long, but an invaluable, series.—RUSH.

### ORIGINAL PAPERS,
### AND
### CASES OBTAINED FROM PUBLIC INSTITUTIONS AND OTHER AUTHENTIC SOURCES.

### *Report of Cases treated at the* ABERDEEN NEW DISPENSARY, *and communicated by* ALEXANDER RAINY, senior Pupil.

#### I. FISTULA LACHRYMALIS.

THIS complaint has been treated at the Dispensary, for the last six months, in a manner somewhat peculiar. When matter has formed in the lachrymal sac, and it has been found impossible to introduce a thin probe through the duct, by the nose, the enlarged sac has been laid freely open, and a tent of waxed sponge kept in the wound till its edges have become callous. By this time, the sac having contracted, the external wound shows no disposition to heal, when the tent is removed. The appearance of the artificial opening thus formed is not at all disgusting; and persons, who were liable to frequent suppurations of the part, have remained free from them after this treatment, a little matter only passing through the artificial opening, after being exposed to cold, or any other cause which increases the flow of tears, or the secretion from the meibomian glands. Of course, in these cases the duct has not been permanently obstructed. The following case was the one which first gave occasion to the above treatment:

Mrs. ——, aged twenty-eight, had a fistula lachrymalis on the left side, for which the os unguis was perforated, to allow the tears to pass by that way to the nose. This was six years since.

Two years ago, the sac of the right side became inflamed, and suppurated, breaking externally. The opening soon healed, but the same process again took place three months after, the disposition to inflammation becoming so great that it now came on after the slightest exposure to cold, and sometimes an interval of only a fortnight occurring between them. She therefore became anxious for the operation : the physician in consultation, however, advised it to be deferred till after delivery, the patient being in the seventh month of pregnancy; and that, in the mean time, the sac should be laid freely open, and a tent kept in the wound. This was done, and the tent worn for two months ; when it was found that the sac had contracted to its natural dimension, and the opening at its edges thickened. The tent, being troublesome, was dropped, and a little clear fluid was occasionally pressed from the cavity, which occasioned no inconvenience; the whole having less appearance of any thing unusual than the other side, where the scar of the last opening was easily observed.

Nothing unusual has occurred since ; the external opening being little observable, and so small as only to admit an Anel's probe.

Three cases have been treated in the same way, but the following is the only one which falls within the quarter's report.

CASE II.—Mrs. M—, ætat. forty-five, July 1st, 1827. Has a tumor in the situation of the lachrymal sac, which is extremely painful, having deprived her of rest for the last forty-eight hours. The eyelids of that side are closed from the swelling, and matter can be perceived by the touch. Says she is obliged to proceed to Glasgow tomorrow, and earnestly begs that something may be done to relieve her. Sac to be opened, and tent introduced, with directions to continue it on board the ship, and for six weeks after.

31st.—Returned two days ago from Glasgow : the sac is now contracted, edges of the wound callous.—Omit the tent.

August 4th.—External opening small, and not to be observed at a short distance. Discharged.

The surgeon consulted in the first case has mentioned that the same result has happened in a case since treated by him on the same principle.

### II. DISEASES OF THE HEART AND AORTA.

CASE I. *Aneurism of the Aorta, bursting externally through the left Mamma.*

July 15th, 1827. — Binks, ætat. forty-eight, a widow, spare habit of body, tall, chest remarkably narrow. Has been subject for the last two years to palpitation, on the smallest exertion.

Nothing unusual, however, was observed till a twelvemonth since, when she complained of difficulty of breathing and excessive paroxysms of coughing. At that time, the pulsation of the heart could only be felt between the fifth and sixth ribs. The carotid artery, however, pulsated very strongly, so as to be seen by the bystanders. Pulse at these times was unusually quick and small. Digitalis and Squill removed the first attack.

In October last, she was again affected in the same way: the palpitations had, however, become more distressing; pulsation could be distinctly felt at the left side of the epigastrium. The throbbing of the carotid was less distinct. Relief was again procured by Squill and Digitalis; thin mucus being expectorated.

Two attacks succeeded, the symptoms being similar, though of longer duration, and yielding less readily to the remedies employed.

For the last five months, she has been constantly attended from the Dispensary. The symptoms have been violent palpitation of the heart, perceived both at the epigastrium and (though less distinctly) below the angle of the left scapula; most distressing sense of sinking on the approach of sleep, and startings after it had come on; cough severe, and still recurring in paroxysms; very scanty expectoration; difficulty of breathing, only occasionally occurring, and relieved readily by a draught of Camphor mixture, with Ether and Opium, which frequently procured good nights.

This state of the symptoms continued till June last, when the fourth and fifth ribs, at their anterior extremity, were perceived to be more prominent than usual, and gave extreme pain on being touched, however gently. From that time to the present, the fulness has increased till the whole of the left has become distended with it. The cough and occasional dyspnœa are settled symptoms, and the mucous rattle more or less distinctly heard by the unassisted ear.

Two days ago, the anterior part of the tumor was observed to be discoloured, when several layers of adhesive plaster were laid over it. This morning, however, the blood burst out at a large opening, which terminated existence in a few seconds. After the appearance of the tumor externally, the pulse had sunk rapidly in strength, and a few small bleedings, which had been employed, were obliged to be discontinued, as an alarming state of faintness occurred immediately on opening the vein. The digestive organs continued healthy to the last, and a small quantity of food was relished on the day of her death. A full meal always produced distressing sense of sinking, and was thrown off on the occurrence of the first paroxysm of coughing.

*Dissection*, 17th July.—Body much emaciated, completely exsanguineous; left mamma purple, with an opening externally, the size of a penny; bulk of mamma greater than in the prime of life. On enlarging the opening into the aneurismal sac, it was found filled with a firm coagulum; a soft membrane lined its internal surface, easily lacerated at the fore part, but firmer towards the bottom of the cavity. Integuments of mamma unusually firm; between them and the sac a layer of coagulated blood, increasing in thickness as it recedes from the outer opening. Cavity, large enough to hold an orange, communicates, by an opening of an inch in diameter, with the interior of the chest, the upper and under edges of which were formed by a breach (by absorption) in the edges of the third and fourth ribs. On removing the sternum and anterior ends of the ribs, so as to leave the connexion of the external disease with the parts within the chest undisturbed; the opening was found to lead directly to the ascending aorta. This artery had its diameter increased to four inches, internal measurement, at this part; at its rise from the left ventricle, its coats are thickened to half an inch, and its diameter one-half greater than usual. Opposite to the fourth rib, its size and thickness is most increased, diminishing rapidly to where it takes its bend, where, after giving off the carotid, it becomes healthy; and, from the left ventricle to its arch, the aorta has bands of a cartilaginous texture, running through its walls in different directions, and on its outer surface appear depositions of coagulating lymph. The heart is considerably enlarged, but particularly its left side, where its coats are very much thickened. Left auricle and ventricle empty. Walls of the right ventricle thinner and paler than natural, and the musculi pectinati of that auricle uncommonly pale, and very thin. The other contents of the chest are healthy. The left lung small. Carotid artery somewhat enlarged in its diameter, and thickened. No other diseased appearances in the viscera.

*Remarks.*—The above case is valuable as illustrating the possibility of a true aneurism, and still more as exhibiting the complication of that form with the false. There was a tumor in the region of the carotid, with pulsation, which it will be perceived had disappeared for some time before death,—*there being some dilatation, but no aneurism of that vessel.* From the heart to the arch of the aorta, that vessel was enlarged greatly in its diameter; its coats much thickened. An opening at its anterior side communicated with an aneurismal sac, lined by several layers of coagulated blood, and which at last burst outwardly. It is surprising that such extensive disease produced so little disturbance in the system.

Case II. *Ossification of the Heart.*

Although present at the opening of the body, this case was only seen twice before death, and that not in the character of medical attendant. The appearances on dissection are given first, and afterwards what is known of the previous history.

August 13th, 1827.—John ——, aged twenty-two. *Sectio cadaveris.*—Body considerably wasted, with slight œdema of the belly and lower extremities; skin and conjunctiva of a deep yellow colour. An incision was made from the top of the sternum to the umbilicus: the integuments being dissected back, and the sternum raised, some slight adhesions were separated which connected the pericardium to the sternum, and which had the appearance of being recent, and were easily torn. From four to five ounces of bloody serum was found in each side of the chest. The pericardium and heart occupied a great part of the left side of the cavity, the left lung being completely hidden in this view. The pericardium extended upwards as far as the top of the sternum, and below its adhesion to the diaphragm, was five inches from side to side, and four from back to front. From the anterior edge of the upper lobe of the left lung, a strong band stretched across to the contiguous side of the pericardium, of about three inches in breadth, and one in length. A similar band joined the inferior lobe of the right lung to the pericardium. The anterior edges of the two lobes of the left lung were also connected by a strong adhesion, which formed a bridge over the sulcus, separating them. Between the back part of the left lung and the ribs opposite their angles, several stringy adhesions were formed, some of them three inches in length. The lungs otherwise were remarkably sound. On opening the pericardium, an ounce and a half of fluid, similar to that in the chest, was removed. The heart exposed was of natural colour, except some yellow spots which penetrated a small way into its substance, near the apex. A little way to the left of the septum, an irregular cavity appeared, about three lines in length, with puckered edges, and having the appearance of the cicatrix of a recent ulcer. The cardiac veins on the surface of the heart were very large, and distended with blood, particularly those on its under surface. On removing the heart, and cutting into the right auricle, the sinus venosus and proper auricle were of a proportionate size to the rest of the organ: its coats, however, were so thin, as to be translucent in the intervals of the musculi pectinati, which were pale, and farther asunder than usual. The tricuspid valve was strengthened by membranous bands, extending from the insertions of the chordæ tendineæ to the parietes of the ventricle. The sides of the right ventricle were thin. The calumnæ carneæ somewhat pale, and formed a close and complicated network, at the bottom of the cavity. On looking at the left auricle, the walls of the sinus venosus were found to be of a dense membranous-looking substance, a line and a half in thickness, and without a vestige of musculi pectinati, or muscular texture; these, however, were present in the proper auricle of this side. The left ventricle presented nothing unusual, till, on removing its valve, an osseous body was found, the size of a horse-bean, imbedded in its parietes, at the root of the valve. At its outer edge, eight ounces of clear serum was found in the abdominal cavity. The liver of natural colour and size, but somewhat firmer than usual. Spleen small, circular, and firm.

The following are the particulars known of the case, the termination of which is related above:

The individual, from his fourteenth year, had been of a very infirm constitution, and was unable to pursue steadily any employment, having in vain tried several. For the last three years he had been subject to palpitations at the chest, on using much exertion, which, from being feeble, and confined to the region of the heart, increased from time to time till the whole of the chest heaved with the motion, and the pulsation was distinguishable at the epigastrium, and left hypochondrium. Three weeks since, anasarca appeared, which rapidly spread over the lower extremities, belly, and back. From the time of its appearance, the palpitations ceased; but the stomach became irritable, rejecting all kinds of food, in any, even the minutest quantity. The appearance of the countenance was anxious in the extreme, and, after the swelling approached the body, the recumbent posture became impossible. Sleep was difficult to be procured, and its approach looked for with terror, startings interrupting it at its commencement. The medical attendants were necessitated, a few days before death, to make small punctures in the scrotum, prepuce, and feet, which drew off a great quantity of water. He sank soon after. During the progress of the complaint, the pulse exhibited great irregularity: latterly, it gave the feel of a fluid passing through a confined tube, and intermitted frequently, defying all attempts to reckon it. He dated the rise of the complaint to a severe pleuritis, which accounts for the old adhesions. Valsalva's treatment kept the disease long in moderation, but latterly he used no depleting measures, and no remedy except digitalis.

Query, was the ossification the original cause of the disease? If so, we presume it must have existed from the commencement. Even at the age of twenty-two, when he died, we imagine the occurrence of such a morbid formation in this organ to be extremely rare.

### III. SCURVY.

#### Case I. *Land Scurvy, successfully treated.*

July 28th, 1827.—George Sim, ætat. fifty-eight, of a bilious temperament, spare habit of body, has several scorbutic blotches in both his legs, and diffuse redness over the instep; ankles swelled, and pitting on pressure; gums soft and ulcerated, with loose teeth: breath fetid; tongue white at the edges, dry and red in the centre: bowels loose; urine clear; pulse eighty-four, weak. Complains of considerable debility, and unwillingness to move. Ill for several weeks. Attributes his complaints to scanty and poor diet. Has used some aromatic sulphuric acid.

Continue the acid.—Habeat Infusi Cinchonæ ℔jss. sumat cyathum bis quotidie.

30th.—Has used a bottle of the infusion since last visit. Says his stomach feels easy; appetite improved; tongue cleaner; bowels regular.—Pergat.

August 2d.—Swelling increased yesterday, having reached as far as the knees. Having been in bed today, the extent of swelling was not discoverable. Cough, with expectoration of mucus in considerable quantity.

Mist. Scillæ coch. ampl. tussi urgen.—Leave off the bark.

7th.—Since last report, has had obstinate costiveness of his bowels, which was treated at first with laxatives, but which required drastic purgatives for its removal. Refuses to resume the bark today, as to it he attributes the costiveness.

Ordered some wine.

9th.—Health considerably restored. Likes the wine, which agrees very well with him. The blotches have completely disappeared from the legs, as also the anasarca.

Continue the wine. A more nourishing diet recommended.

Convalescent.

Case II. *Sea Scurvy, with Effusion into the Chest. Fatal.*

July 15th, 1827.—George Morrison, ætat. thirty-two, seaman, naturally of sanguineous temperament, and muscular figure, returned from a long voyage yesterday. Today has great difficultyof breathing, respiration being carried on by spasmodic and violent action of the intercostal, pectoral, and scaleni muscles; the diaphragm having apparently no motion. Firm, cold swelling of the lower extremities and sides of the belly, yielding to pressure. Blotches on the arms, thighs, and legs, occasionally itching, and bleed slightly on being scratched; gums pale and flabby; countenance pale, and exceedingly anxious; lips purple; pulse 108, soft and compressible; urine scanty and high coloured. Was bled by a midwife last night, which has increased the complaint. Short cough, without expectoration. Can only sit in a raised posture. The cough and difficulty of breathing came on only three days since; blotches of two months' standing. Was able to work a little till a week ago.

Large blister to the breast.—Expectorant pills, and Pulv. Jalap.

16th.—Today thinks he breathes easier; bowels moved; lips less dark coloured. No rest for the last forty-eight hours. Inclined to dose today in his chair, but is prevented by the cough.

Pergat.

Nine P.M.—Having got suddenly worse, again sent for; but, before arriving, he was dead. Attendant states, that he had drank largely of spirits after the morning visit, and that his whole face was of a dark purple for an hour before his death, which she says took place from suffocation.

*Dissection*, 18th.—The opening of the body was deferred yesterday, on account of the unusual warmth remaining in the region of the heart. The face appears tinged and livid. Putrefaction has made considerable progress. On exposing the lungs, they were found gorged with blood, and small tubercles discovered through their substance, apparently in the quiescent state. Twelve ounces of clear serum in the cavity of the chest; four ounces of similar fluid in the pericardium. In the tricuspid and mitral valves, small granular bodies of a cartilaginous texture. No serum in the abdomen. Liver firm; left lobe somewhat enlarged. Other viscera sound.

CASE III. *Sea Scurvy, with Effusion. Fatal.*

July 3d, 1827.—H. C—, ætat. twenty-six, a seaman, returned from a long voyage a few days since. Legs covered with painful blotches, with slight surrounding redness. Great debility; appetite deficient; gums spongy, bleeding on the slightest touch. Frequent thin feculent stools. Has been ill for some weeks.

Ordered vegetable diet, effervescing draughts, with lemon-juice.

13th.—The day after last report, he went to his relations in the vicinity of the town, and called in their physician. Today, however, his father applied at the Institution, giving the following history of him since he was last seen:—The physician who visited him, on seeing the legs, pronounced the case to be syphilis without farther examination, though the patient protested that he never was affected with that complaint. Mercurial pills were ordered; after which, universal swelling took place.

Eight P.M.—Lies with his head supported by pillows. Anasarca over the whole body; lips livid; respiration very short, and accomplished with great effort; pulse 120, extremely weak. Requires his attendant to change his posture very frequently, in hopes of breathing easier. During the visit, after attempting to answer some questions, became insensible, his head sinking down in bed.—Blisters were directed to the breast and inside of the thighs.

15th.—Having died two hours after last visit, the body was opened today, in presence of the physician and a surgeon. The body and limbs were found very œdematous. Twelve ounces of serum in the cavity of the chest. Lungs paler than usual externally: several tubercles existed throughout their substance, but small, and bearing no marks of having been inflamed. A few old adhesions of the lungs to the pleura costalis. Fluid in the abdomen. Some slight appearance of vascularity in the peritoneal covering of the smaller intestines. Other viscera sound.

Query, was not the death in this case occasioned by the free exhibition of mercury, in a constitution completely debilitated, and rendered scorbutic from the effects of salt provisions during a long voyage?

This case demonstrates the impropriety of deciding in complicated cases on a glance at the patient, or on the authority of a single symptom.

IV. SUSPICIOUS DEATH.

*Case of Suffocation, from Carelessness.*

June 8th.—J. B—, six weeks old, was taken fifteen miles into the country by its father and mother, in order to be left at nurse:

the night being cold, it was very closely wrapped up, and the mother, taking it under her mantle, stepped into the gig. During the first part of the journey, the infant cried much, which made the parent, from the idea of her being cold, increase the number of wrappings around her. She was put to the breast, but refused to suck. For the last half hour, the child lay quietly in the mother's arms, and, on handing it out of the vehicle, it was found dead.

The body being exposed, the integuments of the chest and sides of the abdomen were found to be of a dark hue, as were the arms and head. Anterior part of the abdomen of usual colour. A circle round the neck, where there was a deep fold of the integuments, contrasting by its whiteness with darkness of the neck. No lividity, however, in this situation. Head lies forward on the breast. Deep wrinkle between the nose and cheek, from under the eyes to the side of the mouth. Nose wrinkled. Tongue pushed from between the jaws, but not projecting farther than the lips. Mouth open. Thumbs drawn in to the palms of the hand; fingers bent. Knees half bent. Forehead wrinkled. Loins and back part of pelvis discoloured. Cellular substance of the chest vascular. Intestines all over of a vermilion tinge, particularly the small ones, the arteries of which are minutely injected. Vessels of omentum filled with florid blood. Stomach vascular, and filled with a fluid resembling flour paste, but different (at least to appearance) from coagulated milk. Spleen bulky, and of a firm consistence. Upper surface of the stomach more vascular than at the smaller curvature, where it looks healthy; villous coat soft and vascular. Pleura sternalis shows a network of beautifully intermingled red vessels: arteries of the pericardium can be distinctly traced to their terminations on its surfaces. Two drachms of clear fluid in the pericardium. Coats of the aorta and pulmonary artery show their minutest vasa vasorum. Veins of the heart gorged with blood. Thymus gland pale. Lungs of left side natural; lungs in right side bright red, crepitous, floating in water. Bronchiæ filled with red froth. Trachea completely occupied with a reddish mucus, its inner membrane vascular. Scalp vascular. Vessels of brain turgid; dura mater natural in colour, while even its finest vessels can be clearly traced. Three drachms of clear fluid between the tentorium and cerebellum; plexus choroides distended with venous blood; a small quantity of serum in the lateral ventricles. The medulla of the brain, on being cut, exhibits a number of red points.

Query.—Was the doubling of the head on the chest sufficient to produce suffocation, by closing the trachea? Was it owing to the hindrance of the return of the blood from the head, that the encephaloid mass was so vascular? Must it not have been from previous disease, that the abdominal viscera presented the appearances described? Was the general development of the minute vessels greater than can be accounted for on the supposition of the child's having died from suffocation? Was the account given by the parents satisfactory; or could any other cause be assigned for its death? Is the want of a coroner's inquest in this country (Scotland,) an encouragement to guilt?

### V. SUPPURATION OF THE KIDNEY.

*Case of Suppuration of the Kidney; with Dissection.*

August 17th.—Magnus Lister, ætat. fifty, formerly a sailor, latterly a weaver, complains of severe pain in the loins and hypogastrium, with frequent desire to make water, which is red and scanty. Bowels costive; nausea and vomiting. Has a scrotal hernia, which he can return at pleasure. Passed some small stones, of a white colour, by the urethra, a week since. Has occasionally complained of pains in the right side for three years past, on exposure to severe fatigue; has suffered considerably from fever, and disturbance of the digestive organs, with severe pains in the region of the kidneys and bladder, which generally ends in the passing of small calculi and sabulous particles.

Habeat Infusi Lini ad libitum.—Injiciatur enema statim.—Warm fomentations to the loins.—℞. Ol. Ricini ℥j.; Tinct. Opii gtts. L. statim.

20th.—Became rapidly worse after last report. The secretions of urine and fæces having failed, notwithstanding the exhibition of diluents and the free use of cathartic medicines. For the last two days was much distressed with hiccup, and latterly nausea and vomiting of dark coloured matters. Died this morning.

*Dissection*, 22d, seven A.M.—An incision was made from the upper edge of the inguinal ring to the bottom of the scrotum, when the hernia was found to consist solely of omentum, which was more vascular than that in the abdomen, though there was no constriction at the ring. The abdomen being laid open in the usual way, the stomach was found drawn down by the omentum a considerable way; the large curvature opposite the umbilicus. The bowels were of a healthy colour. Some old adhesions were seen between their different convolutions, which separated with the greatest ease. Bladder was found with its coats thickened to nearly half an inch. Its interior was divided into two cavities, each of them capable of containing a walnut, and not admitting of farther distention. The ureter leading to the right kidney was enlarged, so as to admit the fore-finger with ease; its inner membrane was white, pulpy, and thickened. The right kidney was seven inches and a half in length, and proportionately broad; its walls were but half an inch in thickness, and on the lower part of the anterior surface a pit was observed, having all the appearances of a cicatrix; its outer surface had a vascular appearance, but, on being cut into, was paler than usual; its pelvis was found empty. The left kidney was only three inches and a half in length, of the usual figure and colour, excepting at one part where its investing membrane was raised in the form of a vesicle; its walls were of an unequal thickness, at one part being as thin as parchment; on cutting into its cavity, it was filled with pus and pieces of cheesy-looking matter. Body was otherwise sound.

---

